# Cleaning solutions for orthodontic clear aligners: a microbiological and colorimetric analysis

**DOI:** 10.1590/2177-6709.30.2.e2524262.oar

**Published:** 2025-05-30

**Authors:** Paula Cristina Henriques da SILVA, Beatriz Ribeiro RIBAS, Tony Vieira FARIA, Janaína Habib JORGE, Luiz Gonzaga GANDINI, Ary dos SANTOS-PINTO, João Roberto GONÇALVES, Jonas BIANCHI

**Affiliations:** 1 São Paulo State University, Dental School, Department of Dental Science (Araraquara/SP, Brazil).; 2 São Paulo State University, Dental School, Department of Dentistry (Araraquara/SP, Brazil).; 3 São Paulo State University, Dental School, Department of Dental Materials and Prosthetics (Araraquara/SP, Brazil).; 4 São Paulo State University, Dental School, Department of Orthodontics (Araraquara/SP, Brazil).; 5 University of the Pacific, Dental School, Department of Orthodontics (San Francisco, California, USA).

**Keywords:** Clear aligners, Invisalign, Cleaning solutions, Alinhadores transparentes, Invisalign, Soluções de limpeza

## Abstract

**Objective:**

This *in-vitro* study aimed to evaluate the antibacterial efficacy and color changes induced by different chemical agents on the orthodontic aligners.

**Methods:**

The sample consisted of Invisalign® aligners materials and seven solutions were tested: sodium hypochlorite (HYP), sodium bicarbonate (BIC), neutral detergent (DET), chlorhexidine gluconate (CX), white vinegar (VIN) diluted in water (1:3), Corega Tabs® effervescent tablets (COR), and distilled water (DW, control group). The microbiological control was evaluated by analyzing cell proliferation (CFU/ml) and cell metabolism (AlamarBlue® test), and the VITA EasyShade® Advance spectrophotometer was used for color change analysis, using multiple Mann-Whitney tests.

**Results:**

All solutions significantly reduced *S. mutans* biofilm (CFU/mL), except for the control group. The AlamarBlue® analysis showed a significant reduction in cell viability, except for the BIC solution (p=0.183). After 7 days, HYP showed significant color variation, compared to all the other solutions, except for the control group (p=0.095). After 14 days, BIC and CX showed significantly greater color variation than the control group (p=0.007). DET showed a large difference compared to BIC and CX (p=0.007), and a statistically significant difference compared to VIN and COR (p=0.015).

**Conclusion:**

The disinfectant solutions HYP, DET, CX, VIN, and COR significantly reduced the bacterial colonies of *S. mutans* and cellular metabolism. In addition, HYP, BIC, CX, and COR significantly affected the material color.

## RESUMO

**Objetivo:**

Este estudo *in vitro* teve como objetivo avaliar a eficácia antibacteriana e as alterações de cor induzidas por diferentes agentes químicos nos alinhadores ortodônticos.

**Métodos:**

A amostra foi composta por alinhadores Invisalign®, e foram testadas sete soluções: hipoclorito de sódio (HIP), bicarbonato de sódio (BIC), detergente neutro (DET), gluconato de clorexidina (CX), vinagre branco (VIN) diluído em água (1:3), comprimidos efervescentes Corega tabs® (COR) e água destilada (AD) (grupo controle). O controle microbiológico foi avaliado pela análise da proliferação celular (UFC/ml) e do metabolismo celular (teste AlamarBlue®), e um espectrofotômetro VITA EasyShade® Advance foi utilizado para análise de mudança de cor por meio de múltiplos testes de Mann-Whitney.

**Resultados:**

Todas as soluções reduziram significativamente o biofilme de *S. mutans* (UFC/mL), exceto o grupo controle. A análise AlamarBlue mostrou uma redução significativa na viabilidade celular, exceto para a solução BIC (p=0,183). Após sete dias, HIP mostrou variação de cor significativa, em comparação com todas as outras soluções, exceto para o grupo controle (p=0,095). Após quatorze dias, BIC e CX mostraram variação de cor significativamente maior do que o grupo controle (*p*=0,007). DET mostrou uma diferença estatisticamente significativa em comparação com VIN e COR (*p*=0,015).

**Conclusão:**

Soluções desinfetantes de HIP, DET, CX, VIN e COR reduzem significativamente as colônias bacterianas de *S. mutans* e o metabolismo celular. Após algum tempo, HIP, BIC, CX e COR afetam significativamente a cor do material.

## INTRODUCTION

Due to the growing demand for more aesthetic orthodontic treatments, clear aligners have become an excellent alternative to fixed appliances.^1^ Besides aesthetics, it is already well established that periodontal indices such as plaque index, gingival index, probing depth, and bleeding on probing, as well as microbiological indicators such as biofilm formation and risk of white spot lesions are up to 10 times lower in patients who use aligners, when compared to fixed appliances.^2^ ^,^ ^3^

Although these indicators are better in patients who use clear aligners, biofilm formation occurs not only on the teeth surface, but also on orthodontic devices, including removable appliances.^4^ Biofilm accumulation occurs on different surfaces of the aligner. Still, areas with a greater presence of irregularities or more retentive regions, such as cusp tips, favor greater accumulation.^5^ Biofilm accumulation on clear aligners can lead to aesthetic impairment, due to color variation, and unpleasant odors, which can directly affect the patient’s compliance with the treatment.^6^

In addition, several studies have evaluated the color variation of clear aligners when exposed to substances such as Coca-Cola, red wine, and coffee, and all concluded that these substances cause staining of the material.^7^ ^-^ ^9^ Thus, the recommendation is to remove aligners to drink any liquids besides water.

Cleaning substances, such as 1% sodium hypochlorite, 0.5% sodium bicarbonate, and 2% chlorhexidine gluconate are widely used in dentistry to disinfect removable devices, such as prostheses and orthodontic appliances, due to their known action in reducing the presence of microorganisms.^10^ ^,^ ^11^ ^,^ ^12^ However, they have been suggested to cause staining in some materials.^10^

Several other commercial disinfectant solutions, such as Periogard^®^, Cepacol^®^, Ortoform^®^, NitrAdine^®^, and Kukis^®^, have also been tested for microbial control and biofilm reduction.^13^ ^-^ ^17^ However, patients usually search for less expensive cleaning substances, and often alternative disinfecting agents, such as vinegar, baking soda, and soap, are used. 

Therefore, the present study aimed to evaluate the effectiveness of microbiological control based on bacterial colony counting (CFU/ml) of *S. mutans* and cell viability (AlamarBlue^®^ test), and color changes in aligners, using a spectrophotometer, testing seven solutions for 14 days: 0.5% sodium hypochlorite (HYP), sodium bicarbonate (BIC), neutral detergent (DET), 0.12% chlorhexidine gluconate (CX), white vinegar (VIN), Corega tabs^®^ effervescent tablets (COR), and distilled water (DW).

The study aimed to test the hypothesis that low-cost, homemade solutions, such as vinegar and detergent diluted in water, are effective disinfectant solutions for microbiological control and do not cause significant color changes in transparent aligners.

## MATERIAL AND METHODS

### SAMPLE SELECTION

This *in-vitro* study used samples from unused Invisalign^®^ aligners (Align Technology Inc., San Jose, CA, USA) from an upper arch setup sequence. The aligners were cut with a diamond disc, to create the test specimens. For microbiological analysis, the buccal surfaces of the upper right central incisor were used, and the buccal surfaces of the upper left central incisor were used for colorimetric analysis. The sample size was carried out based on similar studies.^7^ ^,^ ^9^ ^,^ ^10^ The cleaning solutions tested were divided into seven groups, as presented in Table 1.

**Table 1:** Characteristics of the disinfectant solutions used.

Acronym	Solution	Commercial name	Manufacturer	Preparation method
HYP	0.5% sodium hypochlorite	Dakken liquid^®^	Dakken Company	Used as supplied
BIC	Sodium bicarbonate solution	CALISUL^®^	Calisul Industries	1 tablespoon in 200 ml distilled water
DET	Neutral detergent solution	YPÊ^®^	YPÊ Company	1 tablespoon in 200 ml distilled water
CX	0.12% chlorhexidine digluconate	Periogard^®^	Colgate-Palmolive	Used as supplied
VIN	Vinegar solution (1:3 dilution)	Castelo^®^	Castelo Vinegars	Diluted 1 part vinegar to 3 parts water
COR	Corega Tabs^®^ effervescent tablets	Corega Tabs^®^	GSK Consumer Healthcare	1 tablet in 200 ml distilled water
DW	Distilled water (control group)	N/A	N/A	Used as supplied

### BIOFILM REDUCTION CAPACITY - COLONY FORMING UNITS COUNT (CFU/ML)

The ability to reduce *Streptococcus mutans* biofilm on the samples was evaluated by analyzing cell proliferation (count of colony-forming units) and cell metabolism (AlamarBlue^®^ test). The *S. mutans* strain used was UA 159, and the method used to count bacterial colonies was similar to the *in-vitro* process used by Akgün et al.^18^

*Streptococcus mutans* were seeded on Brain Heart Infusion Agar (BHI) (K25-610007, Kasvi, São José dos Pinhais, Paraná, Brazil) and incubated at CO_2_ 5%, 37°C for 48 hours. 

Then, 10 colonies of *S. mutans* were inoculated in a tube with 10 mL of BHI broth supplemented with 1% glucose. The tubes were incubated overnight (18 hours), in a 5% CO_2_ atmosphere, at 37ºC; thus, the pre-inoculum was obtained.

After this period, the pre-inoculum was diluted at 1:10 (1mL of the pre-inoculum was added into 9mL of BHI broth supplemented with 1% glucose) to perform the inoculum. Aliquots of 1 mL were taken to the spectrophotometer, where the dilutions’ optical density (OD) was read (600 nm). The tubes were incubated in a 5% CO_2_ atmosphere at 37ºC for the growth of the microorganism until their mid-log phases. After 4 hours of incubation, the OD of *S. mutans* was checked and adjusted to 1x10^7^ CFU/mL.

The negative control group (distilled water) was used to confirm biofilm formation before treating the specimens with disinfectants. Biofilm formation was validated by observing elevated levels of viable microbial counts within the biofilm, measured in colony-forming units (CFUs). Additionally, the RPMI 1640 culture medium exhibited a distinct color change, characteristic of biofilm formation by *Streptococcus mutans*, further confirming the presence of the biofilm.

Before the test, samples from each experimental group (n=21) were washed in running water and disinfected in ultraviolet light in the laminar flow hood for 20 minutes on each side of the test specimen, to eliminate any remaining microorganisms.

The specimens from each experimental group were positioned in a 24-well plate with 750 µL of medium with *S. mutans* and incubated for 90 minutes in an oven at 37°C with 5% CO_2_, for cell adhesion. After the adhesion phase, the contents of the plate wells were removed, and the samples were washed twice with 1500 µL of phosphate-buffered saline (PBS) to remove non-adherent cells. Then, 1500 µL of new RPMI 1640 (Roswell Park Memorial Institute medium) was added and incubated for 48 hours for biofilm formation.

After this period, the specimens were immersed in the disinfectant solutions according to the experimental groups for 15 minutes, except the COR group, which remained for three minutes, according to the manufacturer’s instructions. The specimens were then scraped using a pipette tip for 1 minute, to remove the formed biofilm, and serial dilution was carried out.

After 48 hours of incubation at 37°C with 5% CO_2_, manual colony quantification was performed, and the numbers of colony-forming units per milliliter (CFU/mL) were calculated according to the following formula: 

CFU/mL= number of colonies x 10^n^/q. 

In this formula, n is equivalent to the absolute value of the chosen dilution (from 0 to 4), and q is equivalent to the quantity, in mL, seeded of each dilution on the plates. The procedures were carried out on three different occasions, totaling nine samples of each solution (n=9).

### ASSESSMENT OF CELLULAR METABOLISM USING THE ALAMARBLUE^®^ TEST

The same procedures described above were carried out to analyze the cellular metabolism of the *S. mutans* biofilm using the AlamarBlue^®^ test, which measures cell viability through the activity of mitochondrial enzymes. After the microorganisms’ adhesion phase, the biofilm was washed twice with PBS and, on top of the samples, 1500 μL of new culture medium was placed in each plate well. Then, 150 μL of AlamarBlue^®^ solution was added. The plates were then placed in an oven at 37°C with 5% CO_2_. After 4 hours, the fluorescence of the samples was read using the Fluoroskan Ascent (Fluoroskan Ascent Fl, Thermo Fisher Scientific, Marietta, Ohio, USA) at 560 nm (A560) and 590 nm (A590). The procedures were performed on two separate occasions (n=8).

### COLORIMETRIC ANALYSIS

**Figure 1:**
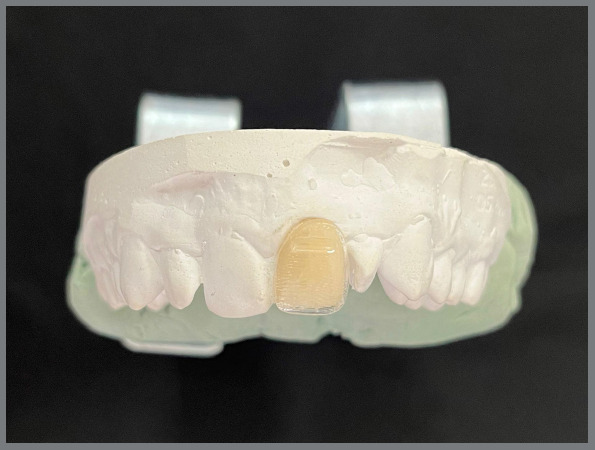
The ceramic crown piece bonded to the plaster model, and the buccal surface of the same tooth of the aligner attached to it.

The VITA EasyShade^®^ Advanced digital spectrophotometer was used for color change analysis. This equipment was developed to determine the color of natural teeth and ceramic restorations. A ceramic crown piece for the upper left central incisor was bonded to the plaster model, to avoid possible displacements. The buccal surfaces of the same tooth were cut out of the aligners and attached to the ceramic piece to measure the color (Fig. 1).

The specimens were divided into seven groups (n=35), according to the disinfectant solutions used. The color of all specimens was measured before immersion in any solution (T0), then, they were kept in artificial saliva and, once a day, immersed in the disinfectant solutions for 15 minutes, except for the COR group which was immersed for three minutes, according to the manufacturer’s instructions. After seven days, a new color measurement was performed (T1) and again after 14 days (T2).

**Figure 2:**
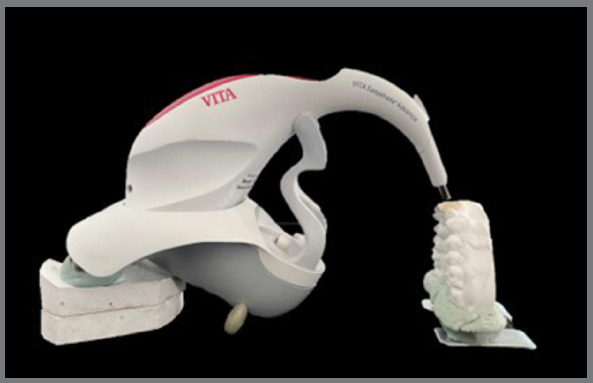
VITA EasyShade^®^ Advance digital spectrophotometer.

The same operator did the measurements under the same lighting conditions, similar to Fotovat et al.^10^ (Fig. 2). Before color reading, all samples were washed with running water, to remove any residue on their surface, and then dried with absorbent paper. Before reading each group, the spectrophotometer was calibrated according to the manufacturer’s instructions. Three readings for each specimen were taken, and the average value was recorded for statistical analysis.

To analyze color variation, the CIEL*a*b* color scale, recommended by the International Commission on Lighting (Commission Internationale de I’Eclairage - CIE, 2004)^17^, was used, which quantitatively evaluates the color characteristics of objects through a mathematical process of three-field colorimetric curve (L*, a* and b*): The color parameter L* represents luminosity (+ light, - dark), a* represents the color scale from red (+) to green (-), and b* indicates the yellow (+) to blue (-) color scale. The total color change (ΔE*) is calculated by the following equation: ΔE* = [(ΔL*)^2^ + (Δa*)^2^ + (Δb*)^2^]^1^ ^⁄^ ^2^, in which, ΔL*, Δa*, Δb* are the differences in L*, a* and b* values ​​before staining (T0) and after (T1 and T2).^9^ ^,^ ^19^ ^,^ ^20^

### STATISTICAL ANALYSIS

The Shapiro-Wilks test rejected the assumptions of normality and homoscedasticity at 5% significance. Then, the Mann-Whitney test verified the research hypothesis. All analyses were performed using the R 4.4 program (2024-04-24, Puppy Cup), with a 5% significance level.

## RESULTS

### BIOFILM REDUCTION

**Figure 3:**
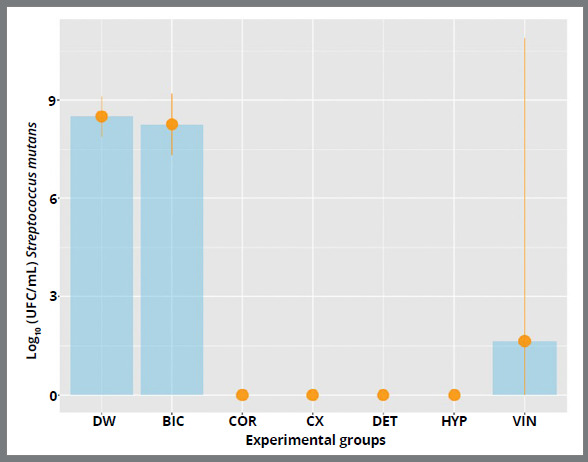
Comparative Mann-Whitney P value for log (CFU/mL) of *S. mutans* ( DW = distilled water; BIC = sodium bicarbonate; COR = Corega tabs; CX = chlorhexidine digluconate; DET = neutral detergent; HYP = sodium hypochlorite; VIN = vinegar ).

All solutions significantly reduced *S. mutans* biofilm (CFU/mL) except for the control group. Groups HYP, DET, CX, and COR showed a 100% reduction (*p*<0.001), as observed in Fig. 3. In comparison, group BIC (p=0.046) (8.2 log) is close to the values obtained in group DW (8.5 log), indicating that this solution is not efficient in the microbiological control of *S. mutans*.

### CELLULAR METABOLISM

**Figure 4:**
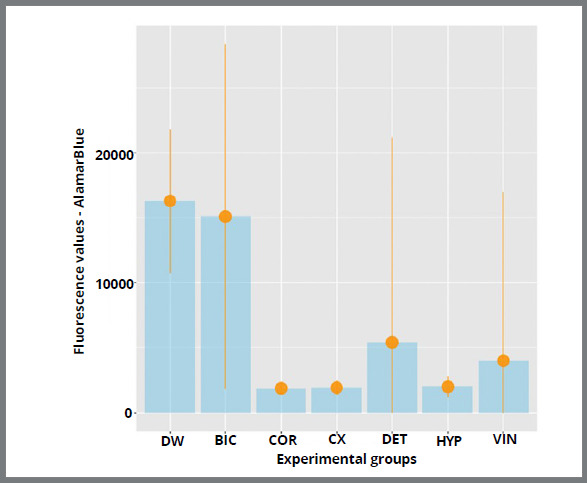
Cell viability analysis - AlamarBlue test ( DW = distilled water; BIC = sodium bicarbonate; COR = Corega tabs; CX = chlorhexidine digluconate; DET = neutral detergent; HYP = sodium hypochlorite; VIN = vinegar ).

The results showed a significant reduction in cell viability for five solutions, except BIC and DW (p=0.183), which were similar (Fig. 4). Groups HYP, DET, CX, VIN, and COR showed similar results (p<0.001), and were more efficient than groups BIC and DW (p=0.183).

### COLORIMETRIC ANALYSIS

The ΔE was calculated using the information obtained with a spectrophotometer (L, a, b) to measure the color variation. For each of the solutions, two deltas were calculated, one comparing the variation after seven days and the other, after fourteen days of immersion. Each specimen was measured three times (T0, T1, and T2) and the average of the values ​​of each time was used as the observational value. Then, the formula mentioned above was used and the summarized values ​​are shown in Table 2. 

**Table 2:** Summary values of color variation.

Solution		Mean	SD	p-value (vs DW 7 days)	p-value (vs DW 14 days)
DW	7 days	1.6	0.915	-	0.8413
	14 days	1.48	0.632	0.8413	-
HYP	7 days	2.746	0.705	0.0952	0.0317
	14 days	2.138	0.95	0.4206	0.2222
BIC	7 days	1.184	0.501	0.5476	0.5476
	14 days	3.152	0.589	0.0317	0.0079
DET	7 days	0.88	0.513	0.3095	0.2222
	14 days	1.166	0.475	0.4206	0.4206
CX	7 days	1.418	0.743	0.7533	0.8413
	14 days	2.756	0.274	0.0317	0.0079
VIN	7 days	1.394	0.702	0.6905	0.8413
	14 days	2.792	1.152	0.1508	0.0556
COR	7 days	1.164	0.829	0.4206	0.8413
	14 days	3.444	1.349	0.0952	0.0465

Bold values indicate statistically significant difference (p<0.005). DW = distilled water; HYP = sodium hypochlorite; BIC = sodium bicarbonate; DET = neutral detergent; CX = chlorhexidine digluconate; VIN = vinegar; COR = Corega tabs.

### AFTER SEVEN DAYS

After seven days, group HYP showed significant color variation, compared to other solutions: BIC, DET, CX, VIN, and COR (p<0.05). However, compared to the control group (DW), there was no statistically significant difference (p=0.095). 

Group DET showed the smallest color variation after seven days (p=0.309), compared to the control group. Groups BIC, CX, VIN, and COR did not show significant differences between themselves or compared to the control group after seven days, suggesting that these solutions do not cause noticeable color changes in a short period (Table 3). 

**Table 3:** Mann-Whitney P value for comparison of color variation after 7-day immersion.

Solutions	DW	HYP	BIC	DET	CX	VIN	COR
DW		0.0952	0.5476	0.3095	0.7533	0.6905	0.4206
HYP	0.0952		0.0079	0.0079	0.0317	0.0317	0.0317
BIC	0.5476	0.0079		0.3095	0.7533	0.6905	0.8413
DET	0.3095	0.0079	0.3095		0.2222	0.2222	0.7533
CX	0.7533	0.0317	0.7533	0.2222		0.8413	0.6905
VIN	0.6905	0.0317	0.6905	0.2222	0.8413		0.8413
COR	0.4206	0.0317	0.8413	0.7533	0.6905	0.8413	

Bold values indicate statistically significant difference (p<0.005). DW = distilled water; HYP = sodium hypochlorite; BIC = sodium bicarbonate; DET = neutral detergent; CX = chlorhexidine digluconate; VIN = vinegar; COR = Corega tabs.

### AFTER FOURTEEN DAYS

After fourteen days, groups BIC and CX showed significantly greater color variation (p=0.007), compared to group DW (control), suggesting that BIC and CX solutions caused a noticeable color change, which increases with immersion time. Group COR also showed a significant color variation, compared to the control group (p=0.046), after fourteen days.

Groups DET and VIN did not show significant differences in color variation after fourteen days, compared to the control group. Group DET showed a large difference when compared to groups BIC and CX (p=0.007), and a statistically significant difference when compared to groups VIN and COR (p=0.015), indicating that the colorless neutral detergent may be more stable in terms of color over time.

Group HYP, although showing significance after seven days, did not maintain this difference after fourteen days, suggesting that the initial color change was not consistent or robust over time (Table 4).

**Table 4:** Mann-Whitney P value for comparison of color variation after 14-day immersion.

Solutions	DW	HYP	BIC	DET	CX	VIN	COR
DW		0.2222	0.0079	0.4206	0.0079	0.0556	0.0465
HYP	0.2222		0.0952	0.0952	0.5476	0.4206	0.2222
BIC	0.0079	0.0952		0.0079	0.22222	0.4206	0.6905
DET	0.4206	0.0952	0.0079		0.0079	0.0158	0.0158
CX	0.0079	0.5476	2.2222	0.0079		1	0.6905
VIN	0.0556	0.4206	0.4206	0.0158	1		0.4633
COR	0.0465	0.2222	0.6905	0.0158	0.6905	0.4633	

Bold values indicate statistics significant difference (p<0.005). DW = distilled water; HYP = sodium hypochlorite; BIC = sodium bicarbonate; DET = neutral detergent; CX = chlorhexidine digluconate; VIN = vinegar; COR = Corega tabs.

## DISCUSSION

The null hypothesis, which stated that low-cost, homemade solutions, such as vinegar and neutral detergent, are ineffective disinfectants and cause significant color changes in orthodontic aligners, was rejected. The present results demonstrated that these solutions significantly reduced *S. mutans* biofilm, with neutral detergent showing minimal color variation over time.

Although orthodontic aligners are removed for eating and hygiene procedures, biofilm formation on their surfaces occurs. Recent systematic reviews revealed that combining mechanical and chemical disinfection methods for removable appliances, whether orthodontic or prosthetic, is more efficient than either method alone.^6^ ^,^ ^21^ ^,^ ^22^

Among the most used chemical agents cited in the literature are chlorhexidine and sodium hypochlorite in different concentrations.^6^ ^,^ ^11^ ^,^ ^13^ ^,^ ^16^ ^,^ ^17^ ^,^ ^22^ These agents are widely used in dentistry due to their excellent antimicrobial action, which can be bacteriostatic or bactericidal, depending on the concentration^11^, which could also be proven in the present study, as both solutions managed to eliminate 100% of the *Streptococcus mutans* colonies from the specimens.

In this study, the cleaning efficacy and colorimetric impact of various solutions on orthodontic clear aligners were assessed. The Efficacy Rate (ER) was calculated by comparing the reduction in *S. mutans* biofilm (CFU/mL) for each solution. Solutions such as sodium hypochlorite, neutral detergent, chlorhexidine gluconate, and Corega Tabs achieved 100% ER, significantly reducing biofilm and cellular metabolism, whereas sodium bicarbonate (BIC) showed limited effectiveness, similar to distilled water (control).

The Stain Level Evaluation (SLE) highlighted the colorimetric effects over time. Solutions like sodium hypochlorite, sodium bicarbonate, chlorhexidine gluconate, and Corega Tabs caused notable color changes, particularly after fourteen days, while neutral detergent and vinegar exhibited minimal changes, maintaining color stability. These findings underscore the importance of selecting cleaning agents that balance microbiological control with minimal aesthetic compromise.

Valentini-Mioso et al.^12^ compared the effectiveness of chemical hygiene protocols for dentures, using solutions of sodium hypochlorite, chlorhexidine, sodium bicarbonate, and distilled water (control), and the results obtained corroborate those found in our study, where both hypochlorite and chlorhexidine acted similarly, promoting a significant reduction of *S. mutans* and biofilm, while sodium bicarbonate showed similar results to the control group.

Regarding the low-cost, homemade solutions tested in the present study, such as neutral detergent and vinegar diluted in water, similar results to those found in previous studies were obtained, such as the study carried out by Aydoğan Akgün^18^, in which the results showed a significant reduction in *Streptococcus mutans* and *Lactobacillus* colonies over four weeks when peroxide and vinegar solutions were used together with brushing. Kiesow et al.,^23^ who also evaluated the microbiological control of some solutions, including neutral detergent and vinegar, in removable partial dentures, pointed out inefficiency against microorganisms typical of bacterial plaque.

Some authors evaluated the effectiveness of cleaning tablets, including Corega Tabs, regarding their antimicrobial action,^24^ ^-^ ^26^ and, as in the present study, the product is effective against “cocci” bacterial species. However, it is recommended to use a mechanical method concomitantly, to completely remove the adhered biofilm.^25^

Some clinicians also recommend that aligners should be brushed with water only; however, in the present study, it was possible to observe that distilled water (control group) did not influence the reduction of bacterial colonies or cell vitality. However, a limitation of the present study was that the specimens were not brushed over time. 

Most of the studies that evaluated the color variation of orthodontic aligners made the comparison based on the consumption of highly pigmented substances such as coffee, Coca-Cola, and red wine.^7^ ^,^ ^8^ ^,^ ^9^ ^,^ ^20^ Few studies were found that evaluated color variation after using different cleaning methods.^27^ ^,^ ^28^ In both studies, seven different cleaning methods were tested, including Invisalign cleaning crystals (Align Technology, Inc.; San José, California); Listerine Cool Mint (Johnson & Johnson Consumer, Inc.; New Brunswick, New Jersey); 2.5% vinegar; 0.5% sodium hypochlorite; 3% hydrogen peroxide; and distilled water. After six months, all solutions showed a significant decrease in light transmittance.

The International Commission on Lighting defines three color spaces for the communication and expression of color, through the LAB system (Commission Internationale de I’Eclairage - CIE, 2004).^18^ The CIELAB color evaluation system quantitatively evaluates the color characteristics of objects, through a mathematical process of a three-field colorimetric curve (L*, a*, and b*): L* is the measure of quantification of luminosity (brightness) in a scale in which black has a value equal to zero, while total light reflection has a value equal to 100; a* measures the amount of red (+a*) and green (-a*); and b* measures the amount of yellow (+b*) and blue (-b*).^9^ ^,^ ^19^ ^,^ ^20^

According to the results obtained in the present study, the solutions of 0.5% sodium hypochlorite, sodium bicarbonate, 0.12% chlorhexidine, and Corega Tabs most promoted color changes. Considering that patients undergoing treatment with aligners consider aesthetics during treatment and that orthodontic retainers are used for the long term, the choice of the cleaning method is extremely important both for microbiological control and to ensure that it does not significantly affect the material’s color.

The present study had some limitations. First, the *in-vitro* model does not fully replicate real-life conditions, such as multiple bacteria, and habits like brushing and eating. The biofilm used only contained *S. mutans*, which does not represent the complexity of oral bacteria. Future studies should include other bacteria linked to gingivitis and periodontitis, for a more realistic analysis. The sample size was also modest, especially for color changes, which may have affected the ability to detect small differences. 

In summary, this study demonstrated that low-cost cleaning solutions, such as vinegar and neutral detergent, are effective in reducing *S. mutans* biofilm without causing significant color changes in clear aligners. These findings highlight the potential of affordable options for aligner maintenance. However, further research, including clinical trials and long-term assessments, is needed to confirm these results and better reflect real-life conditions.

## CONCLUSION

The disinfectant solutions of 0.5% sodium hypochlorite, neutral detergent, 0.12% chlorhexidine (Periogard^®^), vinegar, and Corega Tabs^®^ significantly reduce the bacterial colonies of *S. mutans* and cellular metabolism. The solutions of 0.5% sodium hypochlorite, sodium bicarbonate, 0.12% chlorhexidine (Periogard^®^), and Corega Tabs^®^ can significantly affect the color of the material.

Finally, this study suggests that diluting neutral detergent or vinegar in water, homemade and low-cost solutions, are recommended for cleaning aligners, as they provide excellent microbiological control, without significantly affecting the material color.
